# Communication Skills and Illness Perception Among Non-Japanese-Speaking Patients in Japan

**DOI:** 10.7759/cureus.75403

**Published:** 2024-12-09

**Authors:** Yuko Denda, Honoka Izawa, Hanzhi Wang, Guohua Han, Mariko Santa, Zixuan Cao, Francois Niyonsaba, Ai Noda, Kazuya Hara, Naoko Ono

**Affiliations:** 1 Department of Medical Interpreting, Graduate School of Medicine, Juntendo University, Tokyo, JPN; 2 Faculty of International Liberal Arts, Juntendo University, Tokyo, JPN; 3 Atopy (Allergy) Research Center, Graduate School of Medicine, Juntendo University, Tokyo, JPN; 4 Department of Public Health, Graduate School of Medicine, Juntendo University, Tokyo, JPN

**Keywords:** communication skills, illness perception, language assistance, language barrier, non-japanese-speaking patients, social support

## Abstract

Background: Non-Japanese-speaking patients taking medical treatment in Japan face language barriers and lack of language assistance. Language barriers influence all processes from preventive services to treatment, and insufficient communication can affect patient outcomes. Illness perception, which is related to illness-coping behaviors and self-care behaviors, is an important factor for effective treatment, but no studies have investigated the relationship between communication skills and illness perception among non-Japanese-speaking patients.

Objective: The purpose of this cross-sectional study was to assess communication skills utilizing ENDCORE, to clarify the relationship between communication skills and illness perception among non-Japanese-speaking patients in Japan, and to identify the impact of language assistance on these factors.

Method: A web questionnaire survey was conducted between February and April 2024, targeting 1,904 non-Japanese-speaking patients who had visited medical institutions in Japan. The survey items included basic attributes, purpose of the medical visit, Japanese language proficiency, communication tool, language assistance, communication skills (using ENDCORE), and illness perception (using the Brief Illness Perception Questionnaire; B-IPQ). The Kruskal-Wallis test and Mann-Whitney U test were used to examine factors related to the means of ENDCORE scores and B-IPQ scores, and Spearman’s rank correlation coefficient was used for correlation analysis.

Results: The analysis included data for 354 participants. Participants who received language assistance scored lower on communication skills than those who did not receive language assistance, but they reported more positive illness perception. For factors related to ENDCORE means, statistically significant differences were observed for country of origin, residence qualifications, Japanese language proficiency, communication tool, and language assistance. For factors related to the B-IPQ means, statistically significant differences were observed for sex, country of origin, residence qualifications, communication tool, and language assistance. The correlation analysis of ENDCORE and the B-IPQ items showed that the sense of control a person feels they have over their illness (*r* = 0.201, *p* < .001), the significance of treatment (*r* = 0.215, *p* < .001), and understanding of the illness (*r* = 0.318, *p* < .001) were weakly positively correlated.

Conclusion: The findings suggest that language assistance for non-Japanese-speaking patients can lead to more beneficial communication between them and their physicians and more positive illness perception for those patients. We also believe that maximizing patients’ communication skills will facilitate more appropriate diagnoses and treatments, improve patients’ coping behaviors, and contribute to patient safety and quality of medical care.

## Introduction

The number of foreign residents in Japan at the end of June 2023 was a record high of 3,223,858, an increase of 4.8% from the previous year [1]. The largest proportion of foreign residents was from China at 24.5%, followed by Vietnam at 16.1%, South Korea at 12.8%, the Philippines at 9.6%, and Brazil at 6.5%. Tokyo had the largest number of foreign residents. Since the abolition of border control measures in April 2023, the number of foreign visitors to Japan has been increasing rapidly, reaching 25,066,100 in 2023.

Foreign patients taking medical treatment in Japan face various difficulties. Mizuta et al. showed that foreign patients experienced communication gaps at Japanese medical institutions including “lack of Japanese language proficiency,” “fear of prejudice against foreigners,” “insufficient doctor’s explanations,” and “differences in culture and customs” [2]. Tsuda et al. suggested that among foreign visitors to Japan who have visited Japanese medical institutions, “various anxieties about language and communication may not be fully resolved even when medical interpreters are used” [3]. Hamai et al. reported that insufficient communication can affect patient outcomes, such as delays in treatment and examinations and treatment interruption by patients’ own judgment [4]. Similarly, in other countries, culturally and linguistically diverse patients experience difficulties when taking medical treatment. Al Shamsi et al. showed that language barriers lead to miscommunication between patients and medical professionals, which reduces satisfaction for both parties and diminishes the safety and quality of medical care [5].

It is commonly believed that medical interviews require communication that is different from everyday life. For example, Cambridge reported that the doctor is responsible for changing topics and deciding and controlling the order of conversations in medical interviews and that there is a power imbalance between the patient and the doctor. They also pointed out that it is difficult for doctors and patients to find common ground, and that patients who are unfamiliar with technical terms may feel alienated when doctors use them [6]. When foreign patients communicate with healthcare providers in Japan, they face additional problems such as language and cultural barriers. Therefore, it is important to understand foreign patients’ communication skills and language assistance in medical interviews to avoid or solve ensuing communication problems.

Many studies have been conducted on communication skills. Fujimoto et al. developed a hypothetical model of communication skills called “ENDCORE” and established its reliability. ENDCORE is a model that assesses communication skills and is utilized in various fields. ENDCORE is composed of six factors: “Self-control,” “Expressivity,” “Sensitivity,” “Assertiveness,” “Responsiveness,” and “Regulation.” The language abilities that are the foundation of communication are expressivity and sensitivity, whereas the element that leads to smooth communication is self-control. These three components are considered basic skills. Additionally, ENDCORE is classified into the Expressive System (Expressivity and Assertiveness), the Reactive System (Sensitivity and Responsiveness), and the Management System (Self-control and Regulation). Regarding the communication skills of Japanese university students, Fujimoto et al. showed that the Reactive System skills are higher on average than those in the Expressive System [7]. They also stated, “Because ENDCORE does not include specific cultural situations and contents, it is a multifaceted measure of communication skills that allows cross-cultural comparisons and can measure self-, interpersonal-, social-, and culture-related skills” [8].

A non-Japanese-speaking patient’s perception of their illness is also an important factor for effective treatment. Tanaka et al. showed, for example, that when patients are diagnosed with a serious disease such as cancer, they may request a medical interpreter because of their difficulty in understanding the terminology and their lack of sufficient illness perception [9]. Indeed, Hashimoto asserted that “Illness perception is the preliminary stage in determining the behavior to deal with the illness and is a major factor that determines whether self-care behaviors can be performed effectively or not” [10]. Moreover, Leventhal et al. stated that illness perception is the process of responding to a health threat that generates both cognitive and emotional expressions [11]. According to the common-sense model of self-regulation, Leventhal et al. identified five domains of illness perception: “symptoms and names (identity), expected duration or expected age of onset (timeline), severity of pain and impact on life functions (consequences), infection or genes (internal and external causes), and whether the disease was perceived as preventable, curable, or controllable (controllability)” [12]. Moss-Morris et al. added two domains patients’ understanding of their illnesses (coherence) and emotional responses related to their illness (emotional representations) [13].

The Brief Illness Perception Questionnaire (hereinafter, B-IPQ) is a scale for rapidly measuring illness perception and is composed of nine items that address each of the following areas: “Consequences,” “Timeline,” “Personal control,” “Treatment control,” “Identity,” “Concern,” “Understanding,” “Emotional response,” and “Cause of illness.” It is used to measure patients’ perceptions and emotions regarding their illness [14], and validation of the scale was performed. Asai et al. illustrated that factors related to B-IPQ scores for chronic heart failure patients were whether they had an occupation, the severity of their symptoms, and their psychological conditions [15]. Saudi et al. showed a relationship between blood glucose control and B-IPQ scores in type 2 diabetes patients [16]. While previous studies on illness perception among non-Japanese-speaking patients in Japan have been conducted [17], none have explored the relationship between communication skills and illness perception.

Therefore, we conducted a questionnaire survey of non-Japanese-speaking people who had visited medical institutions in Japan, with the aim of clarifying the relationship between non-Japanese-speaking patients’ communication skills and their illness perception and identifying the impact of language assistance on these factors. The results of this study are expected to foster a better understanding of the need for language assistance among medical professionals, which will lead to improvements in patient safety and quality of medical care.

## Materials and methods

Research participants and survey methods

In this study, we conducted a self-administered questionnaire survey to people who met all of the following criteria: 1) 18 years of age or older whose first language is not Japanese; 2) had visited a Japanese medical institution for themselves or their child’s health issues or injuries; and 3) had given their own voluntary consent to participate in this study. The age limit was set at 18 years old, which took into consideration the age at which patients have sufficient judgmental ability to understand and consent to the explanation; it is also the age at which a person is commonly deemed an adult. The questionnaire was created in four languages, Easy Japanese, English, Chinese, and Vietnamese. A translation company with a proven track record translated English, Chinese, and Vietnamese version questionnaires and then back-translated them to ensure their accuracy.

The survey period was from February to April 2024. We sent emails with the request for participation in the study and the purpose and significance of the study to the heads of the target organizations, which comprised international university student groups, Japanese language schools, local Japanese language classes, international exchange associations, organizations that manage technical intern trainees, and companies. Responses were collected through an online survey using Google Forms from 1,904 study subjects belonging to 20 organizations. We provided information about the self-administered nature of ENDCORE and the B-IPQ. Additionally, we explained the content of the study through face-to-face interviews or an online interview using Zoom. The consent document for the questionnaire survey included the following information: research purpose, type of research participants, survey period, survey method, participation in the study, withdrawal of consent to participate in the study after it has been agreed, handling of personal information, and consultation desk for research participants.

Ethical considerations

The survey request clearly stated assurance of anonymity and privacy of the research participants, the voluntary nature of participants’ research cooperation, and a guarantee that the survey results would not be used for purposes other than the research. A check box was also provided in the consent section of the questionnaire, and checking it was considered consent. The implementation plan for this study was approved by the Research Ethics Committee of the Faculty of Medicine at Juntendo University (Ethics review approval period: December 10, 2021 to December 31, 2031, Approval: December 6, 2023, Approval No: E21-0237-M05).

Survey items

The survey items comprised: basic attributes (age, sex, country of origin, duration of stay, residence qualifications), purpose of the medical visit, Japanese language proficiency, communication tool, language assistance, communication skills (ENDCORE), and illness perception (B-IPQ).

Answer options for length of stay in Japan were: “less than 1 year,” “1-5 years,” “6-10 years,” “11-20 years,” and “21 years or more.” The options for residence qualifications were: “specialized/technical fields,” “specified skilled worker,” “residence status (long-term resident, permanent resident, or spouse of a Japanese national),” “technical intern training,” “student,” “other,” and “no answer.” Specialized/technical fields include professor, artist, religious activities, journalist, highly specialized professionals, business/management, legal/accounting services, medical services, researcher, instructor, engineer/specialist in humanities/international services, intra-company transferee, entertainer, nursing care, and skilled labor. A specified skilled worker means a foreign worker who has a certain level of specialized knowledge and skills in a specific industrial field. Technical intern means foreign intern trainees who receive training in Japanese skills, techniques, and knowledge. The options for purpose of medical visit were: “general outpatient (other than COVID-19 infection),” “emergency outpatient (other than COVID-19 infection),” “second opinion,” “hospitalization/surgery (other than COVID-19 infection),” and “COVID-19 infection.” Japanese language proficiency options were: “I can speak at the same level as a native Japanese speaker,” “I can speak to an extent that does not interfere with work or study,” “I can speak enough to not have any trouble in daily life,” “I can barely speak Japanese.” The communication tool options were: “direct conversation,” “translation device,” “an interpreter.” Language assistance was a choice of “yes” or “no.”

Communication skills were assessed using ENDCORE, a self-administered questionnaire survey that is a simplified version of the ENDCOREs. The Japanese version and English version of ENDCORE were provided to us by the original publishers, and we obtained permission to translate it into Chinese, and Vietnamese. A translation company with a proven track record translated it and then back-translated it to ensure its accuracy. The ENDCORE model comprises 24 items (ENDCOREs) consisting of six factors with four subskills each, and a six-item ENDCORE consisting of only six factors: “Self-Control,” “Expressivity,” “Sensitivity,” “Assertiveness,” “Responsiveness,” and “Regulation.” Answers were scored from 1 to 7 and were calculated using a seven-point scale. A higher total score indicates better communication skills. The contents of each item are shown in Table 1.

**Table 1:** ENDCORE items

Main skills item	Description
Self-Control	I bring my emotion and behavior under good control
Expressivity	I express my thoughts and feelings precisely
Sensitivity	I read other’s feelings and thoughts accurately
Assertiveness	I make a point of my opinion or position to be accepted by others
Responsiveness	I respect and understand other’s opinion or position
Regulation	I work toward others to regulate a good relationship

Illness perception was measured using the B-IPQ, a self-administered survey. The English, Japanese, and Chinese versions of the B-IPQ were provided to us by the original publishers, and we obtained permission to translate them into Vietnamese. As previously mentioned, a translation company with a proven track record translated and back-translated it as well. The B-IPQ consists of nine items, which address: “Consequences,” “Timeline,” “Personal control,” “Treatment control,” “Identity,” “Concern,” “Understanding,” “Emotional response,” and “Cause of illness.” All items except “Cause of illness” were answered on a 0-10 scale and were calculated using an 11-point scale. A higher total score indicates that the patient perceives the illness to be more threatening. A higher score for each item indicates a more negative perception and a higher score for the reversed items indicates a more positive perception. In this study, the free form “Cause of illness” was not included in the analysis for quantitative research. The content of each item is shown in Table 2.

**Table 2:** The Brief Illness Perception Questionnaire (B-IPQ) *Personal control, Treatment control, and Understanding are reversed items, and a higher score indicates a more positive perception.

B-IPQ items	
1.	How much does your illness affect your life? (Consequences)
2.	How long do you think your illness will continue? (Timeline)
3.	How much control do you feel you have over your illness? (Personal control) *
4.	How much do you think your treatment can help your illness? (Treatment control) *
5.	How much do you experience symptoms from your illness? (Identity)
6.	How concerned are you about your illness? (Concern)
7.	How well do you feel you understand your illness? (Understanding) *
8.	How much does your illness affect you emotionally? (Emotional response)
	(e.g., does it make you angry, scared, upset or depressed?)
9.	Please list in rank-order the three most important factors that you believe caused your illness.

Statistical analysis

Descriptive statistics were performed for all variables to show the characteristics of the participants in this study. After performing a normality test for ENDCORE and the B-IPQ, Kruskal-Wallis tests, and Mann-Whitney U tests were performed on the mean using basic attributes (age, sex, country of origin, duration of stay, and residence qualifications), the purpose of medical visit, Japanese language proficiency, communication tool, and language assistance as explanatory variables. Spearman’s rank correlation coefficient was used to analyze the correlation between communication skills and illness perception. Additionally, communication skills were classified into three categories: Expression System (Expressivity and Assertiveness), Reactive System (Sensitivity and Responsiveness), and Management System (Self-control and Regulation), and the correlations between each system and the B-IPQ were analyzed using Spearman’s rank correlation coefficient. The statistical analysis software used was SAS Enterprise Gide 8.4 (SAS Institute Inc, Cary, NC, USA), and a *p*-value less than .05 was considered statistically significant.

## Results

Characteristics of the participants

The basic attributes of the participants are shown in Table 3. The questionnaire was distributed to 1,904 study participants belonging to 20 organizations. Of these, 376 people returned their questionnaires, resulting in a response rate of 20%. Thirteen samples that did not meet the inclusion criteria and nine invalid samples were excluded. Data for 354 participants were analyzed, 190 of whom were male (54%) and 154 were female (43%). The mean age was 29.6 years (SD ± 7.1), with 196 people (55%) in their 20s and 95 people (27%) in their 30s. Country of origin was represented by 145 people (41%) from China and 134 (38%) from Vietnam. Residence qualification results were student for 90 people (26%), followed by specified skilled worker for 68 people (19%), specialized/technical fields for 61 people (17%), residence status for 55 people (16%), and technical intern training for 54 people (15%). The most common length of stay was 1 to 5 years, for 171 people (48%). The most common Japanese language proficiency was: “I can speak enough to not have any trouble in daily life” for 155 people (44%) and “I can speak to an extent that does not interfere with work or study” for 99 people (28%). The purpose of medical visit was general outpatient for 262 people (74%). The communication tool was a direct conversation for 232 people (66%), an interpreter for 71 people (20%), and a translation device for 51 people (14%). Language assistance was used by 122 people (34%) and 232 people reported using no language support (66%). The Cronbach’s alpha coefficients were 0.90 for ENDCORE and 0.72 for B-IPQ.

**Table 3:** Relationship between basic attributes and ENDCORE ^a ^The Kruskal−Wallis test was used to analyze the data, except for language assistance. ^b ^The Mann−Whitney U test was used to analyze the language assistance data. ^c^Specialized/technical fields mean highly skilled foreign professionals. ^d^Specified skilled worker means a foreign worker who has a certain level of specialized knowledge and skills in a specific industrial field. ^e^Technical intern means foreign intern trainees who receive training in Japanese skills, techniques, and knowledge.

	n	(%)	ENDCORE mean	ENDCORE (SD)	ENDCORE median	*p-*Values
Whole sample	354	(100)	29.6	(7.1)	30.0 (6.0-42.0)	
Age						.08
10-19	14	(4)	26.9	(5.6)	26.5 (20.0-42.0)	
20-29	196	(55)	29.2	(7.0)	30.0 (6.0-42.0)	
30-39	95	(27)	29.9	(7.5)	31.0 (6.0-42.0)	
40-49	41	(12)	31.2	(6.9)	32.0 (12.0-42.0)	
50-79	8	(2)	31.4	(5.7)	31.5 (22.0-38.0)	
Sex						.15
Male	190	(54)	29.4	(6.9)	30.0 (6.0-42.0)	
Female	154	(43)	30.2	(7.1)	29.0 (6.0-42.0)	
Others	10	(3)	35.6	(8.5)	26.0 (12.0-42.0)	
Country of origin						< .001
China	145	(41)	30.2	(6.7)	31.0 (6.0-42.0)	
Vietnam	134	(38)	27.6	(7.4)	29.0 (6.0-42.0)	
Others	75	(21)	32.0	(6.2)	33.0 (12.0-42.0)	
Resident qualifications						< .001
Specialized/technical fields ^c^	61	(17)	31.3	(5.8)	32.0 (16.0-42.0)	
Specified skilled worker ^d^	68	(19)	26.0	(7.7)	27.5 (6.0-42.0)	
Residence status	55	(16)	30.2	(6.9)	31.0 (6.0-42.0)	
Technical intern training ^e^	54	(15)	28.7	(7.3)	30.0 (10.0-42.0)	
Student	90	(26)	30.9	(6.5)	31.0 (11.0-42.0)	
Others/No answer	26	(7)	31.7	(6.8)	33.5 (17.0-42.0)	
Duration of stay						.08
Less than one year	58	(17)	30.6	(8.2)	31.0 (6.0-42.0)	
1 to 5 years	171	(48)	29.0	(6.7)	30.0 (10.0-42.0)	
6 to 10 years	86	(24)	29.5	(6.9)	30.0 (6.0-42.0)	
11 to 20 years	26	(7)	29.9	(7.8)	30.5 (12.0-41.0)	
More than 20 years	13	(4)	33.2	(5.1)	35.0 (22.0-39.0)	
Japanese language proficiency						.01
I can speak at the same level as a native Japanese speaker.	53	(15)	32.0	(6.9)	32.0 (10.0-42.0)	
I can speak to an extent that does not interfere with work or study.	99	(28)	30.4	(6.0)	31.0 (12.0-42.0)	
I can speak enough to not have any trouble in daily life.	155	(44)	29.0	(6.6)	29.0 (11.0-42.0)	
I can barely speak Japanese.	47	(13)	27.1	(9.6)	29.0 (6.0-42.0)	
Purpose of medical visit						.56
General outpatient (other than COVID-19 infection)	262	(74)	29.9	(7.1)	30.0 (6.0-42.0)	
Emergency outpatient (other than COVID-19 infection)	18	(5)	28.4	(6.3)	28.0 (16.0-42.0)	
Second opinion	17	(5)	29.1	(8.2)	29.0 (15.0-42.0)	
Hospitalization/Surgery (other than COVID-19 infection)	28	(8)	28.9	(6.6)	30.0 (12.0-39.0)	
COVID-19 infection	29	(8)	28.6	(7.0)	29.0 (12.0-41.0)	
Communication tool						< .001
Direct conversation	232	(66)	30.8	(6.4)	31.0 (6.0-42.0)	
Translation device	51	(14)	27.0	(6.8)	27.0 (12.0-41.0)	
Interpreter	71	(20)	27.6	(8.3)	29.0 (6.0-42.0)	
Language assistance^b^						< .001
No language assistance	232	(66)	30.8	(6.4)	31.0 (6.0-42.0)	
Language assistance	122	(34)	27.3	(7.7)	28.0 (6.0-42.0)	

Factors related to ENDCORE

Significant differences in the mean were found in country of origin (*p* < .001), residence qualifications (*p* < .001), Japanese language proficiency (*p* = .01), communication tool (*p* < .001), and language assistance (*p* < .001). The ENDCORE means for China (30.2) and other countries (32.0) were higher than that for Vietnam (27.6). For residence qualifications, the ENDCORE mean for specified skilled worker (26.0) and technical intern training (28.7) tended to be lower. Those who reported they could barely converse in Japanese had the lowest mean (27.1), and those who directly communicated without language assistance had a higher mean (30.8) than those who received language assistance from a translation device or an interpreter (see Table 3).

ENDCORE was classified into three skill groups: Expressive, Reactive, and Management Systems, and the mean for the Management System (10.3) was higher than that for the Expressive System (9.4) and Reactive System (9.9). This result was observed in both the language assistance and non-language assistance groups (see Table 4).

**Table 4:** ENDCORE classification

	Whole sample (n=354)			No language assistance (n=232)			Language assistance (n=122)		
	ENDCORE			ENDCORE			ENDCORE		
	mean	(SD)	median	mean	(SD)	median	mean	(SD)	median
Overall	29.6	(7.1)	30.0( 6.0-42.0)	30.8	(6.4)	31.0( 6.0-42.0)	27.3	(7.7)	28.0( 6.0-42.0)
Expression System	9.4	(2.6)	10.0( 2.0-14.0)	9.8	(2.4)	10.0( 2.0-14.0)	8.6	(2.8)	9.0( 2.0-14.0)
Reaction System	9.9	(2.6)	10.0( 2.0-14.0)	10.3	(2.5)	10.0( 2.0-14.0)	9.2	(2.8)	9.0( 2.0-14.0)
Management System	10.3	(2.5)	10.0( 2.0-14.0)	10.7	(2.3)	11.0( 2.0-14.0)	9.6	(2.7)	10.0( 2.0-14.0)

Factors related to the B-IPQ

Significant differences were found in B-IPQ means for sex (*p* = .01), country of origin (*p* < .001), residence qualifications (*p* < .001), communication tool (*p* < .001), and language assistance (*p* < .001). The B-IPQ mean for males was low (30.4), and those from Vietnam tended to have a lower mean (22.2) than those from China and other countries. For residence qualifications, the mean values for specified skilled workers (24.6) and technical intern training (24.1) were lower than those for other statuses. A lower mean was observed for those who received language assistance compared with those who directly communicated without language assistance (28.1). Furthermore, the mean for those who used an interpreter was lower on average (24.5) than that for those who directly communicated without language assistance or for those who used a translation device, that is, language assistance through an interpreter tended to have a lower perceived threat of illness (see Table 5).

**Table 5:** Relationship between basic attributes and the B-IPQ ^a^The Kruskal−Wallis test was used to analyze the data, except for language assistance. ^b^The Mann−Whitney U test was used to analyze the language assistance data. B-IPQ: Brief Illness Perception Questionnaire

	B-IPQ Mean	B-IPQ (SD)	B-IPQ Median	*p-*Values
Whole sample	31.3	(14.8)	33.0 (0.0-77.0)	
Age				.06
10-19	28.5	( 9.9)	31.5 (8.0-43.0)	
20-29	30.4	(15.9)	32.0 (0.0-77.0)	
30-39	30.7	(14.0)	32.0 (0.0-60.0)	
40-49	36.2	(12.7)	37.0 (8.0-64.0)	
50-79	38.5	( 7.1)	38.0 (31.0-53.0)	
Sex				.01
Male	30.4	(15.7)	32.0 (0.0-77.0)	
Female	33.9	(10.4)	35.0 (10.0-55.0)	
Others	34.3	(19.0)	29.5 (8.0-58.0)	
Country of origin				< .001
China	36.8	(11.2)	36.0 (0.0-77.0)	
Vietnam	22.2	(14.9)	20.0 (0.0-62.0)	
Others	36.6	(12.7)	37.0 (1.0-64.0)	
Residence qualifications				< .001
Specialized/technical fields	32.8	(12.7)	35.0 (0.0-58.0)	
Specified skilled worker	24.6	(14.8)	27.0 (0.0-54.0)	
Residence status	33.8	(14.4)	34.0 (0.0-64.0)	
Technical intern training	24.1	(17.0)	21.0 (0.0-62.0)	
Student	38.2	(11.6)	37.0 (1.0-77.0)	
Others/No answer	30.6	(12.7)	32.0 (0.0-52.0)	
Duration of stay				.23
Less than one year	34.4	(11.0)	33.5 (1.0-77.0)	
1 to 5 years	29.4	(16.0)	31.0 (0.0-62.0)	
6 to 10 years	31.5	(14.3)	32.5 (0.0-58.0)	
11 to 20 years	32.9	(14.1)	35.0 (8.0-64.0)	
More than 20 years	37.3	(15.3)	40.0 (10.0-64.0)	
Japanese language proficiency				.81
I can speak at the same level as a native Japanese speaker.	30.4	(15.5)	32.0 (0.0-64.0)	
I can speak to an extent that does not interfere with work or study.	31.4	(14.0)	34.0 (0.0-58.0)	
I can speak enough to not have any trouble in daily life.	30.8	(15.2)	33.0 (0.0-64.0)	
I can barely speak Japanese.	33.6	(14.6)	32.0 (0.0-77.0)	
Purpose of medical visit				.34
General outpatient (other than COVID-19 infection)	31.8	(14.7)	33.0 (0.0-77.0)	
Emergency outpatient (other than COVID-19 infection)	31.9	(13.8)	34.5 (0.0-52.0)	
Second opinion	25.2	(14.9)	26.0 (0.0-49.0)	
Hospitalization/Surgery (other than COVID-19 infection)	33.7	(14.9)	34.0 (0.0-64.0)	
COVID-19 infection	27.3	(15.6)	34.0 (0.0-53.0)	
Communication tool				< .001
Direct conversation	32.9	(14.8)	35.0 (0.0-77.0)	
Translation device	33.0	(13.5)	35.0 (0.0-64.0)	
Interpreter	24.5	(13.9)	27.0 (0.0-53.0)	
Language assistance^b^				.01
No language assistance	32.9	(14.8)	35.0 (0.0-77.0)	
Language assistance	28.1	(14.3)	31.0 (0.0-64.0)	

Relationship between ENDCORE and the B-IPQ

The overall means for ENDCORE and B-IPQ were not correlated (*r* = -0.064 and *p* = .23, respectively). Furthermore, each of the ENDCORE skills, which were categorized into three groups (Expressive System, Reactive System, and Management System), and the B-IPQ were not correlated. Specifically, results showed: Expressive System (*r* = -0.045, *p* = .40), Reactive System (*r* = -0.059, *p* = .27), and Management System (*r* = -0.047, *p* = .38) (see Table 6).

**Table 6:** Relationship between ENDCORE and the B-IPQ *r:* Spearman’s rank correlation coefficient; B-IPQ: Brief Illness Perception Questionnaire

	*r*	*p*-Values
Overall	-0.064	.23
Expression System	-0.045	.40
Reaction System	-0.059	.27
Management System	-0.047	.38

Relationship between ENDCORE and each item of the B-IPQ

A Spearman correlation analysis of ENDCORE and B-IPQ items showed statistical correlations. Personal control (“How much control do you feel you have over your illness?”) (*r* = 0.201, *p* < .001), Treatment control (“How much do you think your treatment can help your illness?”) (*r* = 0.215, *p* < .001), and Understanding (“How well do you feel you understand your illness?”) (*r* = 0.318, *p* < .001) were weakly positively correlated (see Table 7).

**Table 7:** Correlation between ENDCORE and each item of the B-IPQ *r:* Spearman’s rank correlation coefficient; B-IPQ: Brief Illness Perception Questionnaire ^a^Personal control, Treatment control, and Understanding are reversed items, with high scores indicating a positive illness perception.

B-IPQ Items	Mean	SD	Median	r	* p-V*alues
Consequence	3.9	(3.1)	4.0	0.047	.37
Timeline	3.2	(3.2)	2.0	0.059	.27
Personal control ^a^	5.7	(3.2)	4.0	0.201	< .001
Treatment control ^a^	6.7	(3.1)	3.0	0.215	< .001
Identity	4.1	(3.1)	4.0	0.103	.05
Concern	5.0	(3.5)	5.0	0.062	.24
Understanding ^a^	6.8	(2.8)	3.0	0.318	< .001
Emotional response	4.2	(3.2)	4.0	-0.038	.48

## Discussion

The purpose of this study was to investigate the communication skills of non-Japanese-speaking patients using a questionnaire, to clarify the relationship between those skills and illness perception, and to identify the impact of language assistance on these factors.

Characteristics of the participants

The majority of respondents came from China (41%) and Vietnam (38%). More than half (60%) were students on study-abroad programs, specified skilled workers, or were doing technical intern training. The majority (82%) were in their 20s and 30s, with a mean age of 29.6 years (SD ± 7.1). According to the Immigration Services Agency of Japan, the largest numbers of foreign residents at the end of June 2023 were from China (24.5%), followed by Vietnam (16.1%) [1], a trend that was similar to the findings of this study. The most common length of stay was 1 to 5 years (48%), and the Japanese language proficiency was reflected in the provided answer options, “I can speak enough to not have any trouble in daily life” (44%) and, “I can speak to an extent that does not interfere with work or study” (28%). According to the Comprehensive Survey of Living Conditions, the data from 2016 showed that teenagers had the lowest rates of subjective symptoms and hospital visits and that these rates increase with age [18]. In other words, age distribution is one of the factors that helps explain why general outpatient visits accounted for 74% of all visits. Moreover, because the reported illnesses were mild and not severe, advanced Japanese language skills may not have been required because the encounters did not involve difficult medical terms or explanations. Indeed, the survey responses, “I can speak to an extent that does not interfere with work or study” and “I can speak at the same level as a native Japanese speaker,” accounted for 43% of participants, which may explain why more than half (66%) of the participants were able to converse directly without language assistance.

Factors related to ENDCORE

For country of origin, the average ENDCORE means for China and other countries were higher than that for Vietnam. This may have been influenced by residence qualifications and Japanese language proficiency, which is the basis for communication skills. Most participants from Vietnam reported residence qualifications of either specified skilled workers or technical intern training. Given that the purpose of specified skilled workers is to support Japan’s labor force, the Japanese language test is required [19]. By contrast, for technical intern training, the Japanese language test is required only for caregiving occupations [20]. Therefore, residence qualifications may affect an individual’s Japanese language proficiency. Aida et al. pointed out that the key is continuing to learn Japanese to improve the communication skills of technical intern trainees [21]. Maeda showed, moreover, that among Filipino technical intern trainees, greater social support resulted in higher Japanese language levels, better mental health, and an attitude of valuing Japanese culture [22]. This indicates the importance of providing Japanese language learning support in communities close to foreign patients, such as in local areas, schools, and workplaces. Additionally, the relationship between linguistic and cultural background and communication skills must be considered. Non-Japanese-speaking patients may be affected by cultural differences in medical interviews-they may struggle to understand the nuances of an unfamiliar language. It is likely that limited knowledge of the Japanese culture and healthcare system can also lead to patient anxiety. Indeed, Ahmed et al. pointed out that communication barriers between immigrant patients and doctors include a lack of language proficiency, hesitation or fear about speaking with doctors, and a lack of trust in the healthcare system [23].

Participants who received language assistance scored lower overall on ENDCORE and for each skill (Expressive System, Reactive System, and Management System) than those who did not receive language assistance. This indicates the need to take into consideration non-Japanese-speaking patients who require language assistance. Morita et al. stated that language barriers affect the entire process from the initial needs to the provision of care, but appropriate treatment, timeliness, and quality of service in the provision of medical care are particularly important [24]. Furthermore, because power imbalances occur in medical interviews, assistance is arguably necessary to help non-Japanese-speaking patients express their thoughts more easily, convey information in a way that is easy to understand, and control emotional reactions caused by miscommunication to build good relationships with those around them. Additionally, because the mean for the Management System was the highest, it is possible that the non-Japanese-speaking patients in this study may have had the ability to adjust and adapt to their surroundings. Providing support such as Japanese language education and interpretation will help improve patients’ Expressive System and Reactive System and ultimately affect overall communication skills.

Factors related to the B-IPQ

We observed a lower B-IPQ mean for those who received language assistance than for those who did not receive language assistance; in particular, those who had an interpreter had a lower B-IPQ mean than those who conversed directly or used a translation device. This suggests that language assistance provided by an interpreter has a positive effect on non-Japanese-speaking patients’ illness perception. Previous studies have also revealed that language assistance affects non-Japanese-speaking patients’ illness perception [17], and the same results were obtained in this study. Communication issues do not only prevent doctors from gathering the information they need to make a diagnosis. If a doctor’s instructions or concerns are not conveyed to a patient, it can also impact effective treatment and patients’ coping behavior. Therefore, interpreters should take into consideration both parties' language and culture during communication between patients and doctors. Translation devices are widely used in Japan and are considered one of the most important methods of language assistance. However, Wang et al. suggested translation devices may not possess “the expertise of medical interpreters to convey complex medical information accurately or maintain cultural sensitivity” [17]. Then, the use of language assistance was a factor in which significant differences were observed among countries of origin. Half of the Vietnamese patients received language assistance, which resulted in lower mean B-IPQ scores and a tendency toward lower perceived illness threat. Regarding residence qualifications, the B-IPQ mean was higher for those who were students and lower for those who were specified skilled workers or technical intern trainees. Kotera et al. stated that “universities, local governments, NPOs, etc., provide health and medical service information in multiple languages to support the health literacy of international students, but it cannot be said that this has been provided sufficiently” [25]. Ishimaru et al., by contrast, showed that 81.2% of the support provided by management organizations for foreign technical intern trainees was focused on medical accompaniment and interpretation [26]. Horimoto et al. reported that support from the workplace or management organization when foreign technical intern trainees receive medical treatment is important for Vietnamese technical intern trainees [27]. For this reason, it is possible that more comprehensive social support, such as management by the host company and interpretation from the management organization, leads to a correct understanding of the illness. Moreover, Pandey et al. noted that language barriers are an obstacle to accessing treatment and preventive services, and they emphasized that language assistance is necessary to give patients an active role and power and to ensure equitable care [28].

Relationship between ENDCORE and the B-IPQ

Communication skills and the overall score for illness perception were not correlated. The B-IPQ questions include the extent to which the illness affects one’s life, whether it is chronic or temporary, the frequency of symptoms, the degree of anxiety, or the type of emotions (e.g., angry, scared, upset, or depressed) caused by the illness; thus, it is possible that the name of the illness and its severity may have influenced the results. In other words, it may not have been possible to show a correlation with overall illness perception based solely on a single factor such as communication skills. Additionally, the system of treatment in which foreign patients’ norms and knowledge are based (e.g., Western medicine or Eastern medicine) may also have influenced their illness perceptions. For non-Japanese-speaking patients, medical interviews in Japan require not only an understanding of medical terminology and medical knowledge but also an understanding of differences in the medical system and treatment environment, thus multiple factors, including cultural background, must be considered when assessing illness perception.

Importantly, nevertheless, correlation analysis between ENDCORE and B-IPQ items showed weak positive correlations for the three items, Personal control, Treatment control, and Understanding, indicating that better communication skills were associated with more positive illness perceptions. Consequently, these findings suggest that communication skills are related to the sense of control a person feels they have over their illness, the significance of treatment, and understanding of the illness. This suggests that communication skills may lead to improved health literacy, which is a determining factor in illness-coping behavior, and adherence through active treatment participation. Therefore, maximizing each person’s communication skills may have an impact on illness perception. Iwata et al. point out that communication challenges for the healthcare provider include nurses’ lack of cross-cultural understanding and a lack of multilingual services. As necessary measures, they suggested providing nurses with learning opportunities about cross-cultural understanding and communication, using visual aids such as charts, pictures, and cards, and using multilingual services [29]. Okamoto et al. state that training for nurses in the use of interpreters and in learning about diversity and cultural humility may enable them to better anticipate and address communication difficulties with foreign patients and their families [30]. Consequently, developing methods and tools for conveying medical information to non-Japanese-speaking patients, as well as providing social support and cultural understanding, is essential for both patients and healthcare providers.

Limitations and significance of this study

This study has limitations. First, we did not provide an analysis of communication skills by educational background, nor an analysis of illness perception by disease name, so the information we extracted for each analysis could be more detailed. Second, the unique focus of this study was non-Japanese-speaking patients, but it did not fully account for cultural differences and differences in healthcare systems at the individual’s level of communication skills and illness perception. Therefore, future research is required to consider these factors. Third, because the study data were collected from 20 organizations, the questionnaires were presented in four languages, which introduced bias into the participants’ country of origin and thus the generalizability of the findings is limited to the surveyed population. However, this study clarified the communication skills and related factors for non-Japanese-speaking patients, reproduced the verification of previous research that language assistance contributes to illness perception, and importantly, observed a relationship for several items in the correlation analysis between communication skills and illness perception; thus, the results of this study can be considered significant.

## Conclusions

The results of this study show that language assistance for non-Japanese-speaking patients leads to more useful communication and more positive illness perception. More specifically, the communication skills of non-Japanese-speaking patients were delineated and our analysis indicates a need for communication strategies based on the characteristics of non-Japanese-speaking patients who require language assistance, and for social support such as interpreters and Japanese language education. The findings also suggest that communication skills may be one factor that leads to appropriate understanding of one’s illness. Therefore, it is necessary to consider specific support for improving the communication skills of non-Japanese-speaking patients. For example, developing communication tools and evaluating their impact on patient outcomes, and implementing pilot programs for language support or cross-cultural communication training in medical institutions and public health centers. These supports not only build good relationships between patients and medical professionals but also make it easier to access treatment and preventive services. Furthermore, they help patients gain a clearer and more appropriate understanding of their illness, which improves their coping behavior and thus provides safer and higher quality medical care.

## Appendices

Questionnaire

English version: https://drive.google.com/file/d/1LM-octrn8g_AEiihQTwjG_YuBM-voMkw/view?usp=sharing

Chinese version: https://drive.google.com/file/d/1JsUlden63nw4JtRztY_4HycsuAEY5Hxf/view?usp=sharing

Vietnamese version: https://drive.google.com/file/d/1pAslEcT9sTH03v5cDRqqqF1Yp5JdPrP0/view?usp=sharing

Easy Japanese version: https://drive.google.com/file/d/168BLhJvD81MnoSk_ON-_zt9v0Hi9xBy7/view?usp=sharing
